# Neutrophil extracellular traps and complications of liver transplantation

**DOI:** 10.3389/fimmu.2022.1054753

**Published:** 2022-11-17

**Authors:** Yanyao Liu, Ping Yan, Yue Bin, Xiaoyan Qin, Zhongjun Wu

**Affiliations:** ^1^ Department of Hepatobiliary Surgery, The First Affiliated Hospital of Chongqing Medical University, Chongqing, China; ^2^ Department of General Surgery and Trauma Surgery, Children’s Hospital of Chongqing Medical University, Ministry of Education Key Laboratory of Child Development and Disorders, Chongqing, China; ^3^ National Clinical Research Center for Child Health and Disorders, China International Science and Technology Cooperation Base of Child Development and Critical Disorders, Chongqing Key Laboratory of Pediatrics, Chongqing, China

**Keywords:** neutrophil extracellular traps, liver transplantation, ischemia-reperfusion injury, acute rejection, thrombosis, hepatocellular carcinoma recurrence, therapeutic targets

## Abstract

Many end-stage liver disease etiologies are attributed to robust inflammatory cell recruitment. Neutrophils play an important role in inflammatory infiltration and neutrophil phagocytosis, oxidative burst, and degranulation. It has also been suggested that neutrophils may release neutrophil extracellular traps (NETs) to kill pathogens. It has been proven that neutrophil infiltration within the liver contributes to an inflammatory microenvironment and immune cell activation. Growing evidence implies that NETs are involved in the progression of numerous complications of liver transplantation, including ischemia-reperfusion injury, acute rejection, thrombosis, and hepatocellular carcinoma recurrence. NETs are discussed in this comprehensive review, focusing on their effects on liver transplantation complications. Furthermore, we discuss NETs as potential targets for liver transplantation therapy.

## Introduction

Neutrophils play a major role in the innate immune response (1), and have a wide range of immune functions, including phagocytosis, reactive oxygen species (ROS) production, lytic enzyme activation, and neutrophil extracellular traps (NETs) production through a process called NETosis (2, 3). NETs comprise chromatin, DNA fibers, and granule proteins. Additionally, NETs are important in treating non-infectious diseases, such as cancer, diabetes, thrombosis, and autoimmune illnesses (4–6). Recent evidence suggests that NETs may contribute to pathological changes after liver transplantation, including liver ischemia-reperfusion injury (IRI), acute rejection, and recurrence of hepatocellular carcinoma (7–9). However, there is little knowledge of the relationship between NET formation and complications of liver transplantation. Herein, we summarize the latest findings that associate NETs with liver IRI, acute rejection, thrombosis, and hepatocellular carcinoma recurrence. We also discuss the potential of NET as a potential therapeutic target in patients following liver transplantation. NET targeting and degradation could be novel promising therapeutic interventions in end-stage liver disease and complications of liver transplantation.

## NET formation

A novel immune defense mechanism known as NETs was discovered in 2004. However, it is difficult to clearly define the specific function of NETs in immune defense (10, 11). With regards to neutrophil pathogenic stimulation, the activation of the signaling pathways, and membrane integrity, the formation of NETs can be classified into three types, namely, lytic, viable, and mitochondrial NET formation (12). Lytic NETs are formed within ten minutes from neutrophil stimulation with phorbol myristate acetate (PMA), lipopolysaccharide (LPS), or IL-8 (13). Several pathways lead to the formation of lytic NETs, including ROS generation in neutrophils that lead to the activation of the enzyme, peptidylarginine deiminase 4 (PAD4). Subsequently, PAD4 converts arginine residues on histones into citrulline, which results in chromatin decondensation (14, 15). In addition, neutrophil elastase (NE) and myeloperoxidase (MPO) are activated and translocated to the nucleus. NE and MPO are also synergistically involved in chromatin decondensation. Likewise, NE can also degrade actin filaments, and block the phagocytosis pathway (16). Single-stranded DNAs and histones are released in the cytoplasm, and form early NETs with antibacterial proteins (e.g., MPO, citH3, NE, and cathepsin G) (17). NET formation requires NADPH oxidase activity and downstream ROS formation (18). Lytic NET formation can be induced by bacteria, fungi and especially chemical stimuli, such as LPS, TNF-α and IL-8 (19). Several *in vitro* studies showed neutrophils formed NET-like structures in response to PMA, LPS, TNF-α and IL-8. In these cells, pretreatment with CI-amidine or use of a PAD4-deficinet line reduced citrullination of histones and NET formation (20, 21).

In viable NET formation, PAD4 is activated by TLR-2 and TLR-4 receptors on neutrophils under different stimuli. For example, bacterial LPS results in the entry of PAD4 into the nucleus to citrullinate the histones, H3 and H4, and unwind DNA strands (22, 23). In contrast to the formation of lytic NET, the PAD4 gene is activated without ROS and does not rupture the cell or nuclear membrane. During the process, the neutrophils are not destroyed, and unwound DNA strands enter the cytoplasm to form early NETs with bacteriostatic proteins. As fascicles, they are exocytosed and released from the cell. Despite the absence of nuclear DNA, neutrophils are still capable of phagocytosing bacteria and killing them (24, 25).

**Figure 1:**
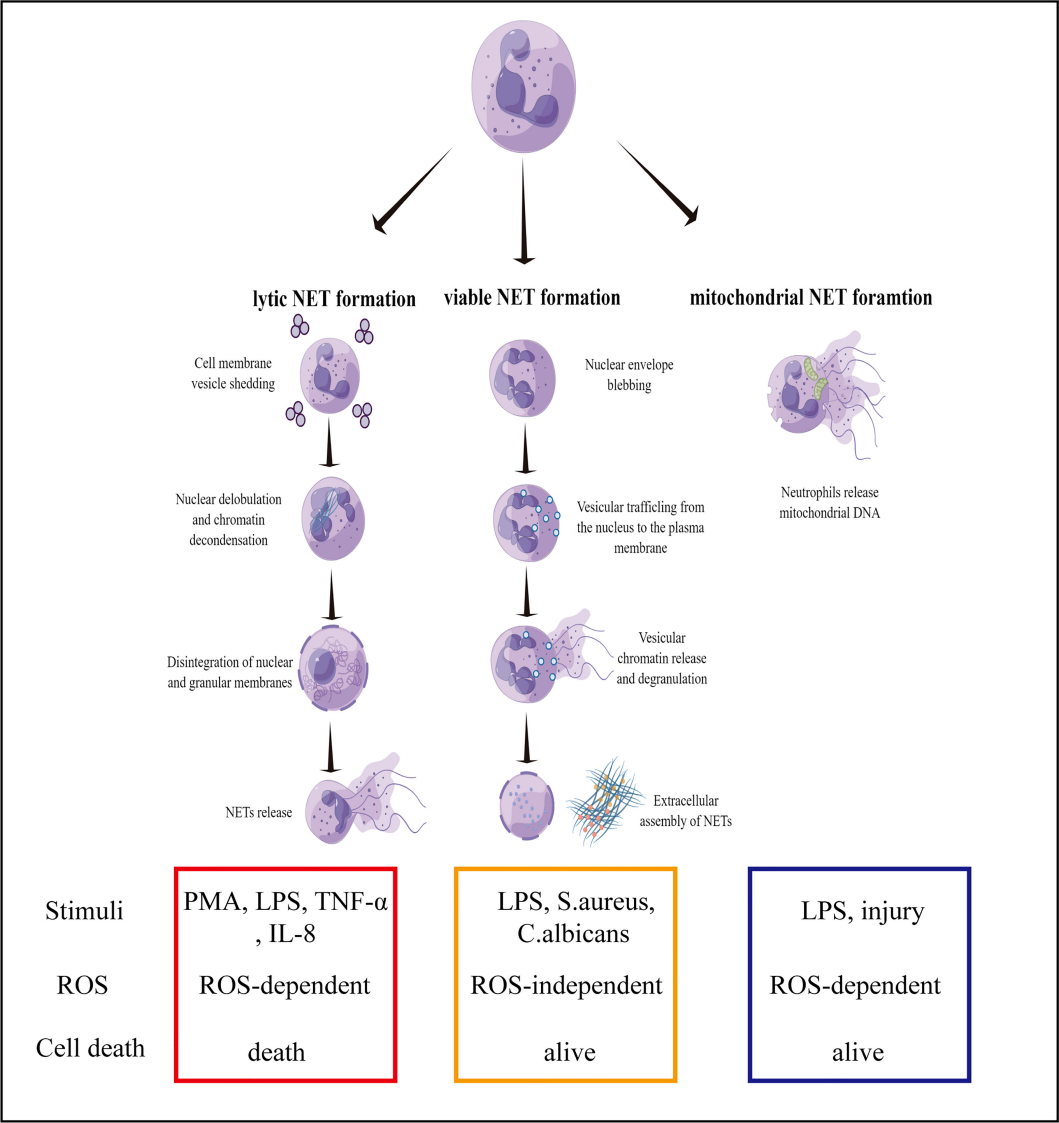
Mechanisms of NET formation. Three mechanisms of NET formation have been described: lytic NET formation, viable NET formation, and mitochondrial NET formation.

The third mechanism describes the formation of NETs with mitochondrial DNA. A previous study demonstrated that eosinophils release mitochondrial DNA after the initial priming with IL-5 or INF-γ, and subsequent LPS stimulation (26). DNA release is ROS-dependent and independent of eosinophil apoptosis. In a subsequent study, mitochondrial NETs were reported to be damaged after the neutrophils were primed with GM-CSF for 20 minutes and then stimulated with LPS for another 15 minutes, which resulted in the release of DNA into the extracellular matrix (27). Neutrophil granular proteins, such as MPO and NE, were also detected in the extracellular matrix with the DNA, but nuclear proteins were not found. It was later reported that NETs contained mitochondrial DNA rather than nuclear DNA sequences (28). Unlike viable NETs, the formation of mitochondrial NETs is ROS-dependent, because the effects of ROS inhibitors and neutrophil deficiency inhibit the release of NETs (29). Recent studies further elucidated the importance of the post-translational modifications of histones, which has a biphasic impact on NETs formation (30). Another study found that the pre-forming protein gasdermin D (GSDMD) plays a key role in neutrophil membrane lysis, nuclear membrane development, and NETs formation (31). However, still unclear about the involvement of PAD4 activation and the presence of mitochondrial DNA in the formation and structure of NETs. To conclude, the mechanism behind the generation and release of NETs requires further investigation (**Figure 1**).

## NETs function

Researchers have demonstrated that NETs have a wide range of efficacy against bacteria, viruses, fungi, and parasites. Several components in NETs, including histones, contain bactericidal and antimicrobial properties (32). NE, a granular protein, can also degrade certain bacterial virulence factors (33). According to prior studies, a fibrous NET structure enhances its bactericidal activity, by either concentrating the antimicrobial molecules into a small area or even serving as a physical barrier against microorganisms (34). Despite NETs’ ability to fight infections, it was soon realized that they were also detrimental to gastrointestinal, liver, and lung inflammations (35, 36). Activated neutrophils co-cultured with enterocyte-like Caco-2 cells revealed that NETs might damage epithelial cells by directly binding to their proteases (37). The researchers also proposed that NETs could facilitate the attachment of enteropathogenic E. coli to the mucosa by causeing damage to the intestinal mucosal barrier (38). Inflammation-associated lung damage and fibrosis are linked to NETs. A recent study revealed postmortem that the four patients who died of COVID-19, each had NETs in their lungs. Airway compartments and neutrophil-rich inflammatory areas of the interstitium contained NETs, while the arteriolar microthrombi contained NET-prone primed neutrophils (39). Murao A, et al. reported that the extracellular cold inducible RNA-binding protein (eCIRP)/TREM-1 interaction and Rho activation are expected to support the development of novel therapeutic molecules able to mitigate inflammation and sepsis by comtrolling NET formation (40).

NETs are considered to be double-edged swords in innate immunity. Because NETs play both an antibacterial and anti-infective role in the early stages of pathogenic microorganism invasion. However, excessive deposition and clearance disorder can lead to inflammation and immune damage to target organs (4, 16, 18, 41). Surgical stress, including liver resection and liver resection and liver transplantation that lead to NET formation. Yazdani et al. found that NET formation was decreased in IL-33 KO mice. IL-33 deficiency protected livers from I/R injury, whereas rIL-33 administration during I/R exacerbated hepatotoxicity and systemic inflammation. *In vitro*, IL-33 mainly released from liver sinusoidal endothelial cells, causes excessive sterile inflammation after hepatic I/R by inducing NET formation (42).

Thrombosis is the formation of a blood clot from the actions of platelets and coagulation factors in the events of vascular damage. A thrombus is formed when coagulation is activated, and fibrinolytic activity is decreased, thereby causing vasculitides to block and disrupt the blood supply to the tissues (43–45). Recent studies have reported the presence of neutrophils and NETs in the thrombus of humans and mice (46). In addition, NETs have been found to stimulate both internal and external coagulation pathways that promote thrombosisby providing a scaffold for the deposition of fibrinogen, platelets, von Willebrand factors, and erythrocytes (47). NETs also promote the deposition of thrombogenic substances. As platelets aggregate and become activated in NETs, the histones interact with fibrinogen, TLR2, and TLR4, to generate thrombin (48). In mouse models, DNase I can effectively prevent intravascular microthrombosis, which suggests a key role of NETs in thrombosis (49). However, another study argued that NETs promote thrombosis through their DNA and histone components instead of the deposition approach (50). That said, further studies are required to fully understand the promoter role of NETs in thrombosis.

Over the past few years, NETs have attracted increasing attention due to their essential role in innate immunology and thrombosis. However, there is also evidence that NETs play a pro-tumorigenic role in cancer (51). A growing number of studies are looking into the potential diagnostic and prognostic values of circulating NETs (52). The deposition of NETs promotes tumor cell proliferation, immunosuppression, and cancer-associated thrombosis. In addition, NETs can accelerate metastasis by contributing to epithelial-to-mesenchymal transition. NETs collect and multiply circulating tumor cells, resulting in tumor cell intravasation and micrometastases (53). At the same time, post-operative infections can increase NETs deposition, which exacerbates the recurrence and progression of post-surgical cancer (54). Considering their integral role in cancer, NETs could be potential therapeutic targets to inhibit tumor cell proliferation, metastasis, and thrombosis.

With deepening research in the field of NETs in liver transplantation, multiple studies discovered that DAMPs, including HMGB1 and histones or superoxide released during liver IRI, related in NETs formation. TLR-4 and/or TLR-9-myeloid differentiation primary response protein signaling pathways stimulated by HMGB1 and histones, respectively, are thought to exacerbate liver IRI (7, 55, 56). Our study founded that NETs promote kupffer cell M1 polarization and intracellular translocation of HMGB1 aggravating liver IRI even cause acute graft rejection following liver transplantation (57).

## NETs detection

Whilst the importance of NETs has been highlighted in innate immunity, it is a challenge to detect NETs due to their heterogenous and acellular structure (58). Moreover, primary human neutrophils cannot be transfected for mechanistic interrelation studies, further complicating NET-related studies (59). Besides that, NETs must be distinguished from cell-free DNA (cfDNA), which originates independently of necrosis and apoptosis (60). Hence, it is crucial to discover NETosis markers and develop quantitative detection strategies that are sensitive and specific, particularly towards lytic NETs. Immunoconfocal microscopes are commonly used to detect NETs *via* immunocytochemistry and immunohistochemistry. Several groups have recommended co-localizing at least three key NET components (i.e., extracellular DNA, NE, and histones) for the accurate detection of NETs. This co-localization helps to differentiate NETs from dead or dying cells that release DNA (61). SYTOX Green dye stain is more specific than DAPI for the detection of NETs in a mixture with extracellular DNA (62, 63). Despite the simple concept, the methodology is not well-developed. There are several challenges to this, such as the need for researchers to manually evaluate the presence of neutrophil-derived proteins and DNA, difficulty in quantifying the formation of NETs, and controversial reported analytical techniques (64). H3cit, MPO and NE are considered as NETs-specific biomarker. Thus, those markers can be used for the ELISA-based detection of NETs (65).

To improve the detection of NETs *in vitro*, two types of flow cytometry methods (i.e., image-based and cell-appendant) have been developed using antibodies against major NET components (66). For example, Zhao et al. used multispectral imaging flow cytometry to identify the swelling of the nuclei in NET-neutrophils as a potential marker for NETosis (67). According to Gavillet et al., NETs simultaneously express both MPO and citrullinated histones on their surface, and these molecules can be detected by flow cytometry (68). In another study, Cichon et al. introduced a novel method to detect NET formation *in vivo via* intravital microscopy (69). Recently, it was reported that CDr15 dye stain was impermeable to cell membranes and emitted strong fluorescent signals when bound to the extracellular DNA of NETs. When compared to SYTOX Green, CDr15 showed lower background fluorescence and higher specificity towards NETs. This was supported by the successful detection of NETs stained with CDr15 in formaldehyde-fixed tumor specimens (70). These novel approaches highlight the promising future developments of NET detection technologies. With advancing technology in NETs detection, accumulating evidence demonstrated that NETs may be a potential biomarker of inflammation and autoimmune diseases to reflect the degree of tissue damage and inflammatory conditions (71). A study reported that the serum levels of NETs changed dynamically during severe fever with thrombocytopenia syndrome (SFTS) progression. High NETs levels were strongly associated with multiple pathological processes and predicted severe illness in patients with SFTS (72). Another study found that serum levels of NETs can provide a picture of systemic inflammatory state and thereby estimate risk for HCC recurrence after surgery. The research of NETs detection technology have important clinical implications for both treatment and biomarker discovery (73).

## NETs and end-stage liver disease

NETs can also play a pivotal role in liver diseases such as acute liver failure, alcohol-associated liver disease, non-alcoholic steatohepatitis (NASH), liver cirrhosis, and hepatocellular carcinoma (HCC) (59, 74, 75). Globally, liver cirrhosis ranks among the top ten leading causes of death (76). A recent study by Zenlander et al. suggests that the level of plasma markers for NET formation correlates to the severity of liver dysfunction in patients with liver cirrhosis and HCC. A comparison between patients with liver cirrhosis and HCC showed that there was no significant increase in the plasma NET markers (77). Another study demonstrated that a higher rate of complications such as recurrent infections, may occur in liver cirrhosis patients with deficient NETs (78). NASH is becoming the most prevalent chronic liver disease in Western society due to its increasing prevalence (79). According to findings by Van der Windt et al., NETs may be involved in the protumorigenic inflammatory environment in NASH. The strategies used to eliminate NETs may also reduce the risk of HCC in NASH (78). Another study proved that NETs promote regulatory T cell activity through metabolic reprogramming in NASH. In other words, therapeutic targeting of NETs and regulatory T cells can prevent the development of HCC in NASH patients (80).

Acute liver failure (ALF) is a life-threatening condition, that is caused by a variety of factors, including viral infections and drug-induced liver damage (81). A clinical study involving 676 patients with ALF reported that patients with ALF had 7.1 folds higher levels of cfDNA and 2.5 folds higher levels of MPO-DNA complexes, as compared to healthy controls. The levels of cfDNA did not correlate with the 21-day transplant-free survival, but they were higher in more severe cases. This finding suggests that NET formation is a contributing factor to disease development (82). Another study of ALF in mice reported the pathogenic role of NETs in ALF. The management of ALF could be improved by regulating the levels of miR-223/NE and NETs (83).

**Figure 2:**
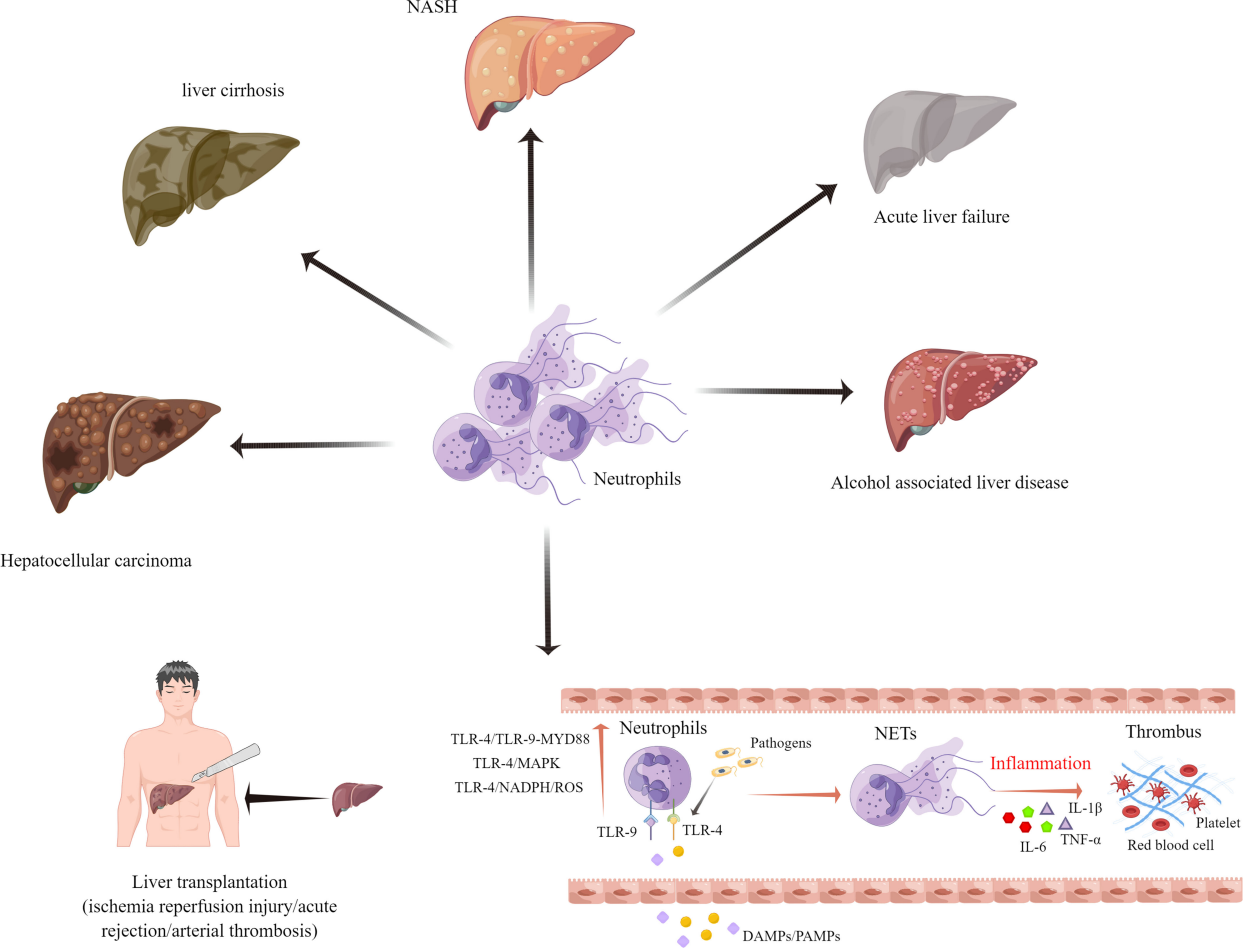
Neutrophil extracellular traps have been implicated in the pathophysiology of several different end-stage liver diseases.

There is also growing evidence that the presence of NETs in a cancer inflammatory microenvironment promotes HCC cell proliferation (53). Researchers found that neutrophils isolated from patients with HCC produced more NETs *in vitro*. The presence of elevated MPO-DNA was associated with increased mortality after liver surgery in HCC patients (84, 85). The oncogenic role of NETs in HCC has been preliminary confirmed. However, the specific mechanisms of NETs in portal vein tumor thrombus and HCC recurrence after surgical resection require further investigations (**Figure 2**).

## NETs and IRI

End-stage liver disease patients benefit from liver transplantation. However, liver IRI is a major cause of liver failure and graft loss associated with liver transplantation (86, 87). In clinical studies, ischemia-reperfusion-related tissue injury accounts for approximately 10% of early graft failures and is a major contributor to both acute and chronic rejection (88). Ischemia livers produce lesser ATPs due to lower oxygen levels. As ATP is low, ROS cytokinesis, vasoactive agents, and adhesion molecules are produced, which can aggravate the damage (89). As a result of ROS generation, the concentration of intracellular calcium increases, and the pH changes, leading to apoptosis and necrosis (90). An important component of liver IRI is inflammation, and neutrophils play an important role in the events leading to liver injury after reperfusion. The excessive activation and recruitment of neutrophils during reperfusion contribute significantly to the pathogenesis of IRI. A neutrophil induces liver injury through a multistep process that involves neutrophil activation, vasculature transport, and migration across the endothelium (91–93).

**Figure 3:**
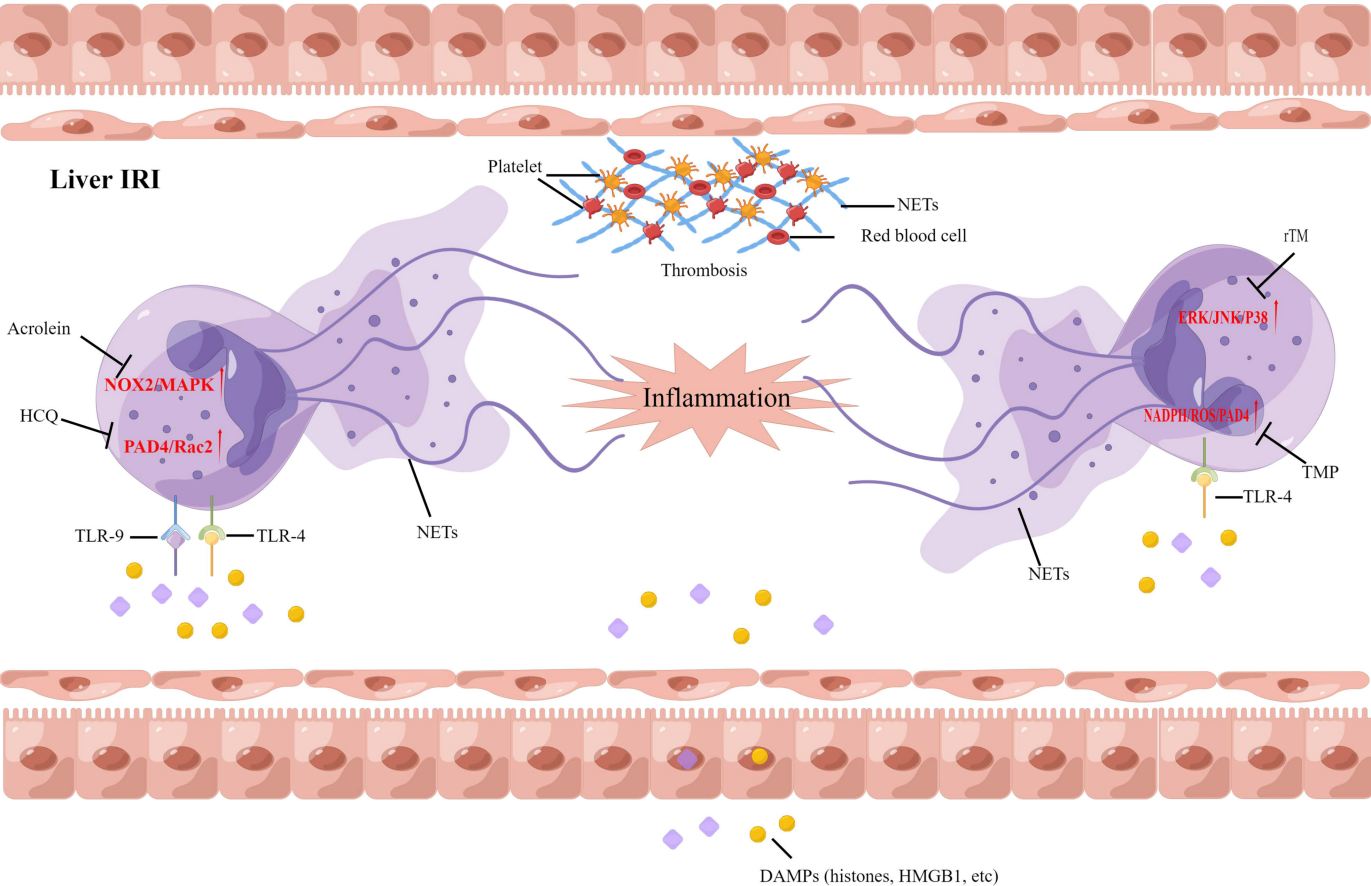
Neutrophil extracellular traps have been implicated in the pathophysiology of liver ischemia-reperfusion injury following liver transplantation. DAMPs, damage-associated molecular patterns; HCQ, Hydroxychloroquine; TMP; Tetramethylpyrazine, rTM; recombinant human thrombomodulin.

As more studies on the functions of NETs emerge, it is implicated that NETs may contribute to the pathogenesis of hepatic IRI. Histones and high mobility group box 1 (HMGB1) proteins, commonly associated with tissue damage, are released from damaged hepatocytes, and this activates TLR4 and TLR9 to induce NETs formation. A recent study suggested that NADPH-mediated superoxide production initiates NETs formation after IRI. Pretreatment with allopurinol and N-acetylcysteine was found to decrease NETs formation and liver injury after ischemia injury in mice (94). Neutrophils and NETs were found in the liver from ischemia-reperfusion mice models, and both were negatively correlated with histidine-rich glycoprotein (HRG) expression. Supplemental HRG treatment inhibited neutrophil infiltration and NETs formation in livers to alleviate liver IRI (95). Zhang et al. also correlated the presence of NETs with hepatic IRI, and hydroxychloroquine could alleviate hepatic IRI by inhibiting NETs formation in hepatic ischemia-reperfusion mice models (55). One study suggested that acrolein can cause the release of NETs in the liver during IRI and slow the recovery rate of a post-operative liver. In patients with chronic hepatic disorders, targeting NOX2 and P38MAPK signaling could inhibit the formation of NETs, and improve the survival and function of the post-operative liver (56). In our study, we found that tetramethylpyrazine (TMP), a compound extracted from *Ligusticum wallichii* Franchat, has the potential to improve liver functions and alleviate hepatic IRI. Furthermore, TMP inhibited NADPH oxidase activity, thus inhibiting the formation of NETs in rats after liver transplantation. We provide the first evidence of a synergistic effect between TMP and diphenyleneiodonium to alleviate hepatic IRI (96). We further examined the effect of recombinant human thrombomodulin (rTM) on liver transplantation in a rat model, focusing on the TLR-4/MAPK axis. Our data illustrated that NETs independently contribute to hepatic IRI, and rTM treatment mitigated neutrophil infiltration and suppressed NETs formation after liver transplantation (97). Although these results suggest that antioxidant treatment can protect against liver IRI *via* the attenuation of NETs formation, the therapeutic benefits of NETs inhibition should also take into account the possible complications in immunocompromised individuals after transplantation (**Figure 3**).

## NETs and acute rejection

Many individuals with end-stage liver disease around the world benefit from liver transplantation (98). According to a recent publication, the 5-year survival rate of grafts and patients after a liver transplant was 72.8 and 76.1%, respectively. Acute rejection (AR) is a common complication after liver transplantation, that affects about 25 to 50% of patients (99). There is evidence that immunosuppressive agents can reduce the rates of acute rejection, but immunosuppressive treatments can also decrease the survival rate of patients (100, 101). Total bilirubin, alanine aminotransferase, and aspartate aminotransferase are commonly used clinically to monitor the liver function of liver grafts (102). Furthermore, the levels of immunosuppressive drugs in the blood can be monitored to predict the risk of AR (103). However, standard laboratory tests are inefficient for detecting AR, in terms of time and specificity (104, 105). Hence, the identification of therapeutic targets and early diagnostic strategies for AR should be the focus of future research (106). Recently, donor-derived cell-free DNA (dd-cfDNA) in AR is attracting increasing attention as a diagnostic biomarker (107). Allograft injury releases dd-cfDNA into the patient’s serum, which makes it a good biomarker to evaluate the condition of allografts and the possibility of rejection (108). A study was conducted by Schutz et al. that measured the levels of dd-cfDNA in 107 patients with liver transplantation. Patients with AR had the highest percentage (29.6%) compared with those healthy controls (3.3%) (109). A more recent study suggested that dd-cfDNA is even more sensitive than conventional transaminases to detect AR (110).

**Figure 4:**
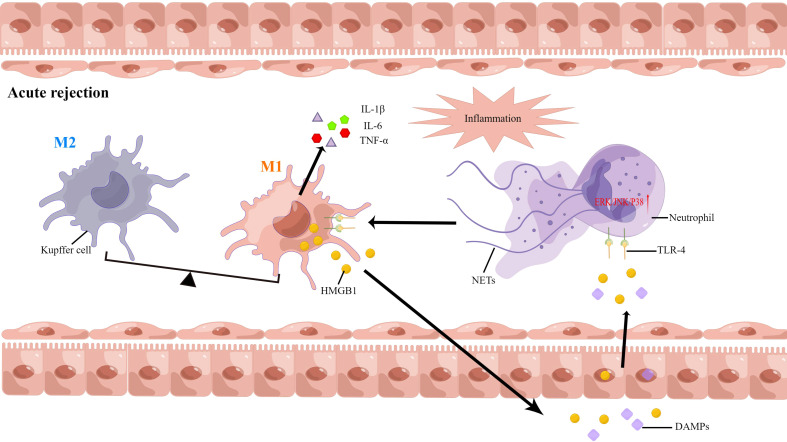
Neutrophil extracellular traps have been implicated in the pathophysiology of acute rejection following liver transplantation.

Extracellular DNA is the most important component of NETs, and neutrophils are generally activated in the AR. However, there are insufficient studies to assess the correlation between NETs formation and AR after liver transplantation. As such, we have conducted some studies in this area (8, 15). Serum samples obtained from 13 liver transplant individuals were analyzed, and we found that the levels of NETs were elevated. During recovery, the levels of NETs decreased gradually and then stabilized. The levels of NETs increased following AR diagnosis, and decreased following treatment with oral rapamycin, in three patients. Next, the serum NETs were measured in liver transplant patients with AR. The levels of NETs in patients undergoing liver transplantation were positively correlated with liver enzymes and the incidence of AR. Our findings revealed that AR is influenced independently by NETs and that NETs subsequently induced kupffer cell M1 polarization and intracellular translocation of HMGB1. On the other hand, HMGB1 activates the TLR-4/MAPK signaling pathway, which causes NETs formation. This positive feedback loop between neutrophils and kupffer cells further amplifies the inflammatory signals and graft injury. Additionally, NET inhibitors combined with immunosuppressive agents may offer a novel treatment option for AR (57). NETs are a potential novel target for AR diagnosis and treatment (**Figure 4**).

## NETs and arterial thrombosis

Hepatic artery thrombosis is the most common vascular complication, that may lead to non-functional liver graft and acute liver failure, following liver transplantation (111, 112). A significant proportion of this is seen in patients with recurrent biliary tract infection or asymptomatic biliary leakage with liver dysfunction (113). Thrombosis after liver transplantation has a high incidence rate and poor prognosis in children undergoing liver transplantation because it is difficult to diagnose in the early stages (114). It is well-recognized that neutrophils and platelets act as first responders to injuries and infections (115). As part of their host defense mechanisms, neutrophils promote blood coagulation by increasing fibrin deposition and limiting the spread of infections (32). NET-fibrin interactions prevent bacterial invasion into the surrounding tissues of the liver microvasculature, while their disruption promotes bacterial dissemination throughout the body (116). A dysregulation or excessive stimulation of the vasculature may lead to pathological thrombosis. Therefore, neutrophils play a pivotal role in regulating thrombosis through several mechanisms (117).

NETs have been recently identified as new DNA-based components involved in the formation of blood clots and thrombosis (118). Platelets, red blood cells, and platelet adhesion molecules adhere to NETs *via* a scaffold, which promotes thrombosis (119). Additionally, many of the scaffold components can also stimulate platelet activation and blood coagulation (6). In addition, NETs can stimulate both intrinsic and extrinsic coagulation, primarily through the serine proteases in neutrophils. Endothelial cells are highly cytotoxic to the histones, H3 and H4, and that platelets can aggregate because of these histones (5). In comparison to venous thrombosis, arterial thrombosis is more common in acute events, as a result of thrombus shedding in acute myocardial infarction (AMI), ischemic stroke, and acute arterial embolism (120). According to Riegger et al., NETs were present in the thrombus of stroke patients and atherosclerotic plaques of patients with atrial fibrillation (121). Another study found that NETs were more prevalent in newly formed coronary thrombi than in older ones and that both myocardial infarction and ST-segment elevation were positively associated with the level of NETs in the coronary thrombi (122). The surgical stress response from liver resection and transplantation can aggravate the deposition of NETs in the liver, and platelets activated with NETs can produce a systemic procoagulant state, leading to immunothrombosis and remote organ injury (123). A mouse model with liver IRI was found to significantly increase both circulating platelet activation and platelet-neutrophil aggregation. NETs and platelet-rich microthrombi were found in the microvasculature of injured organs after liver surgery, and the inhibition of NETs with DNase reduced immune thrombosis and organ damage (124). Although the key role of NETs in immune thrombosis and its related mechanisms have been reported by a large number of studies, the findings on the regulation of coagulation and immune thrombosis after liver transplantation are still lacking. Hence, it is necessary to explore the role and specific mechanisms of NETs in immune thrombosis after liver transplantation, for better management of the condition.

## NETs and hepatocellular carcinoma recurrence

Hepatocellular carcinoma (HCC) and end-stage liver diseases have widely benefited from liver transplantation (125, 126). However, HCC recurrence is one of the main causes of mortality in HCC patients who undergo liver transplantation (127, 128). It has been established that certain tumor characteristics can lead to the recurrence of HCC, including the concentration of alpha fetoproteins, tumor diameter, macrovascular invasion, and extended orthotopic liver transplantation criteria (129). A recent study found that both pre-operative serum hepatitis B viral DNA and pre-operative prognostic nutritional index can potentially be used to predict HCC recurrence after liver transplantation (130, 131). However, these studies all had small sample sizes or were conducted retrospectively and lack of molecular-biological investigations. Despite these advances, the mechanism behind the high HCC recurrence rates remains a mystery, and that these biomarkers have clear limitations.

Recently, NETs have been detected in various cancer samples (i.e., breast, liver, and gastric cancers) and metastatic tumors. In tumor development, NETs play an important role in cancer immunoediting and immune-cell interactions (132–134). Research suggests that NETs activate dormant cancer cells, which causes tumor recurrence (135). Furthermore, HMGB1 is also involved in NETs formation in TME by interacting with TLR4, and this releases excessive inflammatory cytokines (136). NETs also promote cancer invasion and migration, which exacerbates tumor aggressiveness (137). It is well known that the degradation of matrix proteins inhibits the immune system of the host, which is one of the mechanisms of tumor evasion (53). NETs-associated proteinases activate matrix metalloproteinases to induce tumor-associated macrophages, which stimulate the release of pro-inflammatory factors (i.e., IL-8, IL-1β, and TNF-α), eventually leading to immune escape and tumor metastasis (138).

Tumor metastasis is the main cause of cancer mortality, and neutrophils are involved in this process (139). Multiple studies reported that NETs trap circulating cancer cells and release proteases, which results in tumor metastasis and proliferation (35, 140). Najmeh et al. demonstrated that β1-integrin can induce NET-related entrapment of circulating lung carcinoma cells, resulting in cancer development and metastasis (141). These results were supported by Cools-Lartigue et al., where circulating lung carcinoma cells were found to be encapsulated in NET DNA conglomerates in a murine model. It was also shown that circulating “NETs-cancer cells packages” seeded in the liver, produced micrometastases within 48 hours and secondary hepatocellular carcinoma after two weeks (51). A retrospective analysis found that high level of NETs predicted shorter recurrence-free survival and overall survival. Serum levels of NETs as a biomarker pre-surgery can help identify patients with a higher risk for HCC recurrence (73). Another study showed that HCC is capable to stimulate NETs enriched in oxidized mitochondrial-DNA, which are highly pro-inflammatory and pro-metastatic (142). Cheng Y et al. demonstrated that combination of NK cell adoptive therapy and hydrogel-based delivery system can destruct NETs and prevent post-resection and post liver transplantation HCC recurrence (143). NETs play an integral role in cancer invasion, transport, and transendothelial migration according to a multilevel model, especially in HCC recurrence should be further studied.

## Potential therapeutic targets for NETs

In various diseases, NETs play the role of pathogenic drivers, thus making them attractive therapeutic targets. Studies have found that the levels of NETs correlated with the survival of cancer patients (142, 144). However, the risks of using NETs as therapeutic targets should also be evaluated. Targeting NETs would increase infection susceptibility, considering the protective role of NETs against severe infectious diseases (58). A study reported that mice with deletions of PAD4 were more vulnerable to bacterial infections (145). According to another study, PAD4 knockout may protect mice from polymicrobial sepsis-induced septic shock (146). Therefore, the potential risk of targeting NET formation may be determined by the type of disease and immune status of the organism. Another major risk of NETs degradation is the release of NETs-derived DNA and histones, which may trigger inflammation. Currently, therapies targeting NETs can be segmented into two categories: degradation/destabilization of NETs, and the inhibition of NETs formation.

The degradation of NETs has already been extensively studied. Research found that DNase I was capable of partially lysing NETs, and that tPA and DNase I could synergistically initiate thrombolysis (122). The use of DNase I as a treatment in mice suffering from thrombosis was also effective at preventing recurrent stroke, myocardial infarction, and deep vein thrombosis (49). However, further research is required to determine whether DNase I degradation of NETs would increase inflammation and risk for thrombosis. It has also been suggested that treatment with low molecular weight heparins (LMWH) can reduce NETs formation (5). Some researchers reported that histones could be dissociated from the chromatin backbone of NETs *via* heparin therapy and that LMWH can inhibit PMA-induced NETs formation (147). According to a study, the therapeutic use of heparin to treat NET-associated pathologies reported the opposite effect. Lelliott, et al. showed that heparin induced NETs formation *in vitro*, in the absence of PAD4 (148). Two independent studies reported that heparin-induced thrombocytopenia-related thrombosis was caused by NETs (149, 150). These contradictory results, as well as the potential side effects and risks, suggest the need for further investigations.

**Figure 5:**
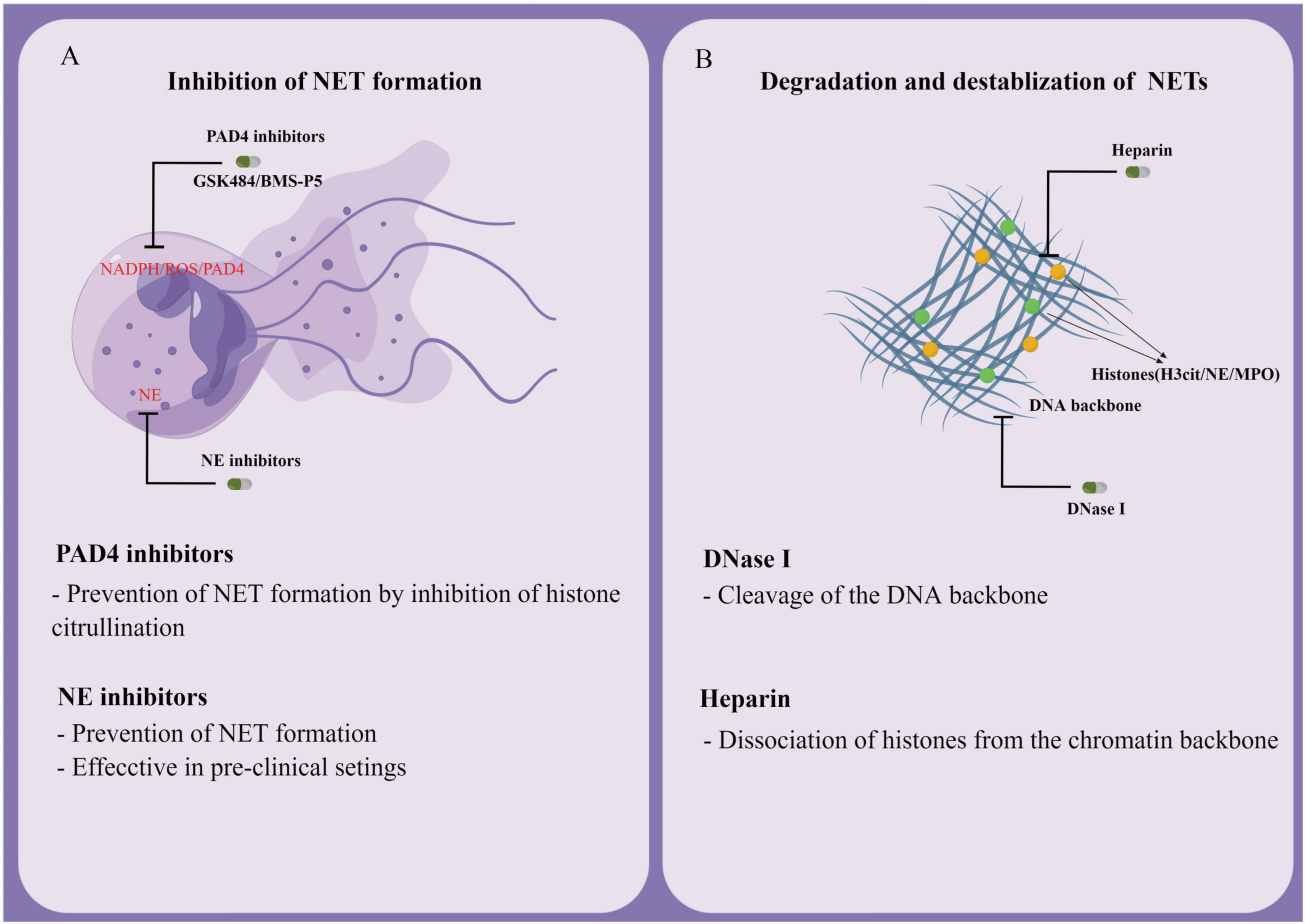
Potential therapeutic targets for NETs. **(A)** Inhibition of NET formation. **(B)** Degradation and destabilization of already formed NETs.

Another strategy to target already formed NETs is to interfere with their formation. Cloamidine, a pan PAD inhibitor, was found to inhibit the expression of PAD4, which subsequently prevents NETosis (151). Another potential advantage is that PAD4 deficiency in mice does not affect bacteremia during polymicrobial sepsis. The efficacy of GSK484 (developed by Glaxo Smith Kline) and BMS-P5 (developed by Bristol-Myers Squibb) in inhibiting NET development and suppressing associated diseases have now been confirmed by several *in vitro* and *in vivo* studies (152, 153). As a potential therapeutic target, NE inhibitors have proven effective in inhibiting NETs formation (51). For example, it was demonstrated that Sivelestat (an inhibitor of NE) inhibited NETs growth in mice with lung carcinoma (154). Antibodies are also known to prevent the formation and release of NETs in several inflammatory conditions, owing to their action toward citrullinated proteins (**Figure 5**) (155).

## Conclusion and future perspectives

There is increasing evidence showing that NETs contribute to ischemia-reperfusion injury, acute rejection, thrombosis, and the recurrence of hepatocellular carcinoma. There is also potential for NET-related molecules as biomarkers and as targets for therapeutic intervention in complications of live transplantation. With further study, NETs is promising to provide a vast number of innovative applications in liver transplantation. There is an urgent need for the development of new methodologies to accurately detect NETs formation, considering the limitations of current methods. In addition, NETs detection should be standardized to ensure consistent results from comparative studies by different research groups. Thus far, strong evidence has shown that NETs might induce inflammation and tumor immune escape in ischemia-reperfusion injury and recurring hepatocellular carcinoma. Further research is required to understand the pathogenicity of NETs in liver transplantation. Neutrophils and NETs play a pivotal role in immune defense, and their potential as therapeutic targets warrants further study. A long-term safety assessment is also needed to assess the benefits and risks of NET-inhibition treatment. As an emerging field within liver transplantation, the relationship between NETs and the postoperative complication of liver transplantation also requires further investigation.

## Author contributions

All authors listed have made a substantial, direct, and intellectual contribution to the work and approved it for publication. All authors contributed to the article and approved the submitted version.

## Funding

This work was supported by the National Natural Science Foundation of China (No. 81873592 and No.82170666).

## Conflict of interest

The authors declare that the research was conducted in the absence of any commercial or financial relationships that could be construed as a potential conflict of interest.

## Publisher’s note

All claims expressed in this article are solely those of the authors and do not necessarily represent those of their affiliated organizations, or those of the publisher, the editors and the reviewers. Any product that may be evaluated in this article, or claim that may be made by its manufacturer, is not guaranteed or endorsed by the publisher.
